# Higher resuscitation guideline adherence in paramedics with use of real-time ventilation feedback during simulated out-of-hospital cardiac arrest: A randomised controlled trial

**DOI:** 10.1016/j.resplu.2021.100082

**Published:** 2021-01-30

**Authors:** Rasmus Meyer Lyngby, Lyra Clark, Julie Samsoee Kjoelbye, Roselil Maria Oelrich, Annemarie Silver, Helle Collatz Christensen, Charlotte Barfod, Freddy Lippert, Dimitra Nikoletou, Tom Quinn, Fredrik Folke

**Affiliations:** a Copenhagen Emergency Medical Services, Copenhagen, Denmark; b Kingston University and St. Georges, University of London, London, United Kingdom; c ZOLL Medical Corporation, Chelmsford, MA, USA; d Herlev Gentofte University Hospital, Copenhagen, Denmark

**Keywords:** BLS, Basic life support, ALS, Advanced life support, CONSORT, Consolidated Standards Of Reporting Trials, CPR, Cardiopulmonary resuscitation, EMS, Emergency Medical Services, ERC, European Resuscitation Council, OHCA, Out-of-Hospital Cardiac Arrest, SGA, Supraglottic airway, sROSC, Sustained return of spontaneous circulation, TBI, Traumatic brain injury, VQI, Ventilation Quality Indicator, Real-time feedback, Ohca, EMS, Ventilation

## Abstract

**Objectives:**

To investigate whether real-time ventilation feedback would improve provider adherence to ventilation guidelines.

**Design:**

Non-blinded randomised controlled simulation trial.

**Setting:**

One Emergency Medical Service trust in Copenhagen.

**Participants:**

32 ambulance crews consisting of 64 on-duty basic or advanced life support paramedics from Copenhagen Emergency Medical Service.

**Intervention:**

Participant exposure to real-time ventilation feedback during simulated out-of-hospital cardiac arrest.

**Main outcome measures:**

The primary outcome was ventilation quality, defined as ventilation guideline-adherence to ventilation rate (8–10 bpm) and tidal volume (500−600 ml) delivered simultaneously.

**Results:**

The intervention group performed ventilations in adherence with ventilation guideline recommendations for 75.3% (Interquartile range (IQR) 66.2%–82.9%) of delivered ventilations, compared to 22.1% (IQR 0%–44.0%) provided by the control group. When controlling for participant covariates, adherence to ventilation guidelines was 44.7% higher in participants receiving ventilation feedback. Analysed separately, the intervention group performed a ventilation guideline-compliant rate in 97.4% (IQR 97.1%–100%) of delivered ventilations, versus 66.7% (IQR 40.9%–77.9%) for the control group. For tidal volume compliance, the intervention group reached 77.5% (IQR 64.9%–83.8%) of ventilations within target compared to 53.4% (IQR 8.4%–66.7%) delivered by the control group.

**Conclusions:**

Real-time ventilation feedback increased guideline compliance for both ventilation rate and tidal volume (combined and as individual parameters) in a simulated OHCA setting. Real-time feedback has the potential to improve manual ventilation quality and may allow providers to avoid harmful hyperventilation.

## Introduction

Close to 700,000 people suffer sudden out-of-hospital cardiac arrest (OHCA) in the United States and Europe each year and fewer than 15% of those treated by Emergency Medical Services (EMS) personnel survive to hospital discharge.^1^, ^2^ In addition to high quality chest compressions, ventilations must be delivered to the patient at an appropriate rate and volume to optimise the flow of oxygenated blood.^3^ Myocardial perfusion, cardiac output, and blood flow to the brain have all been shown to decline with hyperventilation, while cerebral and coronary perfusion pressures significantly increase when ventilation rates are reduced.^4^, ^5^, ^6^, ^7^, ^8^ However, one study have reported no compromise of haemodynamics during hyperventilation.^9^

For patients with secured airways, the European Resuscitation Council (ERC) recommends delivering 1 breath every 6 s (10 breaths per minute) with a tidal volume between 500 and 600 ml per breath while continuous chest compressions are being performed.^3^

While reports of healthcare providers performing CPR outside of guideline recommended parameters can be found in the literature,^5^, ^6^, ^7^, ^10^ some studies have demonstrated that chest compression quality can be improved by providing real-time feedback during resuscitation.^11^, ^12^, ^13^, ^14^, ^15^

Increases in survival and favorable neurological outcome have also been reported with implementation of training and real-time CPR feedback^11^ but which intervention, or combination of interventions, that contribute to improved outcomes remains unclear.

Given the challenge of attaining guideline recommendations for ventilation in OHCA, the impact of hyperventilation on physiology and the improvement in chest compression quality with real-time feedback, this study investigates whether real-time visual ventilation feedback improves manual ventilation quality during resuscitation in a simulated cardiac arrest scenario using a novel flow sensor (AccuVent™) technology. We hypothesize that the use of real-time visual ventilation feedback during CPR will improve ventilation quality delivered by prehospital providers in a simulated OHCA setting.

## Methods

### Study design

**Fig. 1:**
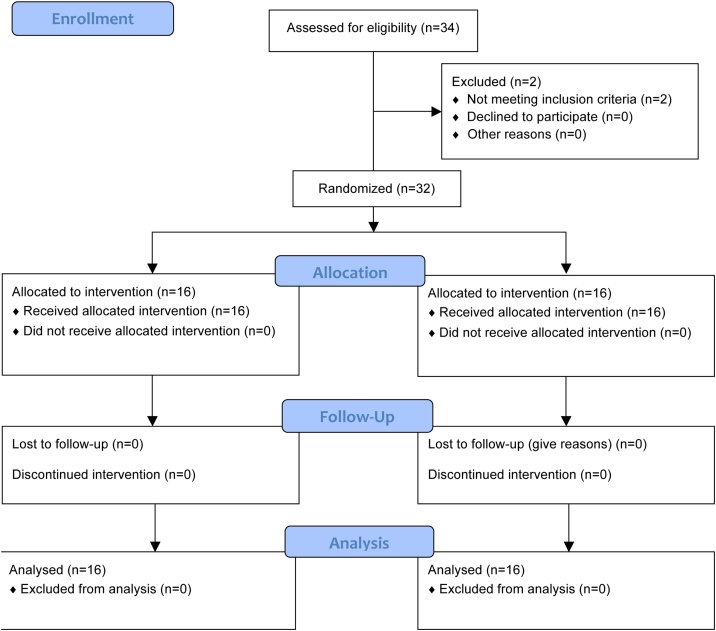
Participant flow. CONSORT 2010 Flow Diagram.

This study was conducted as a non-blinded randomised controlled simulation trial in an out-of-hospital environment. Participating EMS crews were allocated 1:1 to either intervention (real-time ventilation and chest compression feedback) or control (real-time chest compression feedback only) (Fig. 1). Data were collected from 26 August 2019 to 30 August 2019 and written consent was obtained from participants prior to inclusion.

### Intervention

**Fig. 2:**
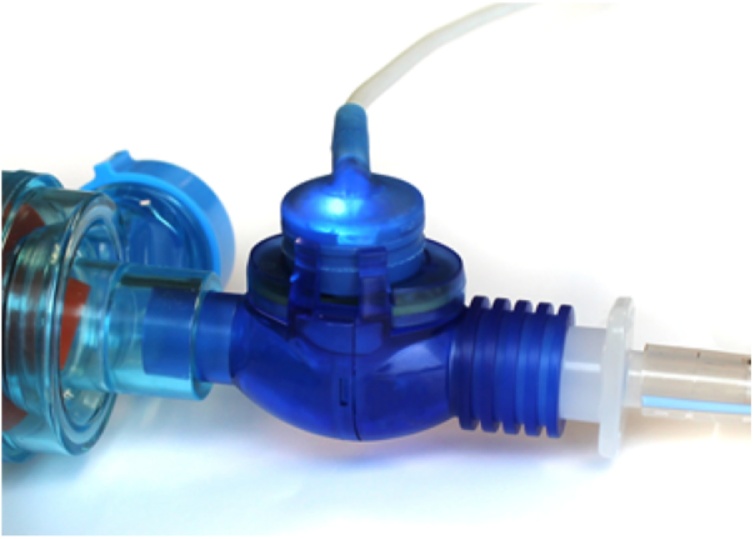
AccuVent™ flow sensor.

AccuVent (ZOLL Medical Corporation, Chelmsford, MA) is a disposable differential pressure-based flow sensor that is designed to measure the delivered ventilation rate and tidal volume and provide breath-by-breath feedback to the rescuer (Fig. 2). The sensor is connected between the bag and the mask or endotracheal tube. The sensor is connected to a reusable cable, which is attached to the X Series monitor/defibrillator (ZOLL Medical Corporation, Chelmsford, MA).

**Fig. 3:**
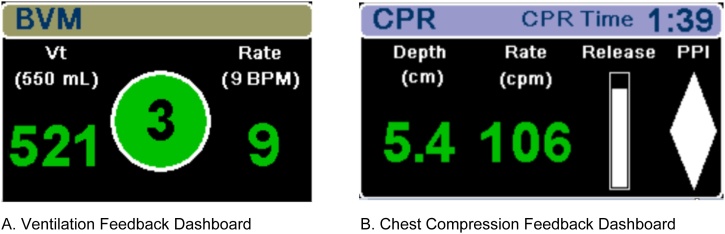
Ventilation and chest compression feedback dashboard. BVM = Bag valve mask, Vt = tidal volume, BPM = breaths per minute, CPR = Cardiopulmonary resuscitation, cm = centimeter, cpm = compressions per minute, PPI = Perfusion Performance Indicator.

The ventilation feedback dashboard features a breath-by-breath numerical value for ventilation rate (breaths per minute) and tidal volume (ml), a countdown timer, and a ventilation quality indicator (VQI). Numerics for ventilations delivered within specified target range are displayed in green, while ventilations outside of target range are displayed in yellow. The countdown timer is positioned inside of the VQI, which counts down to prompt the user to deliver a breath based on the target rate setting. When a breath is delivered, the VQI will gradually fill until target volume is reached, then the graphic will change to green if both numerics are in target or yellow if one or both are out of target (Fig. 3). This technology enables the user to adjust their manual ventilation delivery in real-time during patient care. The ventilation feedback dashboard can be combined and displayed with the chest compression feedback dashboard (Fig. 3).

### Participants and procedure

Participants were recruited among on-duty basic life support (BLS) and advanced life support (ALS) paramedics from Copenhagen EMS. All participants were experienced in using real-time feedback for chest compressions during OHCA but had no previous exposure to the AccuVent sensor or the ventilation feedback dashboard. Ambulances were selected by the dispatch-center based on unit location (their proximity to the study site) and availability (no calls at the given time).

Upon arrival at study-site (Herlev University Hospital and Hvidovre Hospital, Copenhagen, Denmark), crews were given a standardised introduction containing information regarding the research conducted, the simulated OHCA scenario, and timeframe for their participation. They were then screened for inclusion prior to signing informed consent to participate. Participants were required to be trained BLS or ALS providers and perform CPR as a part of their job description. Furthermore, participants were to confirm exposure to at least one OHCA within the last 6 months or attendance at CPR training within the calendar year. Active first aid instructors, physical limitations, and known pregnancy were exclusion criterias. Prior to randomisation demographic information (age, gender, level of training, years in EMS) were recorded.

### Randomisation

Participants were assigned to the intervention (real-time ventilation and chest compression feedback) or control (real-time chest compression feedback only) group by a simple blocked randomisation procedure conducted on-site (Fig. 1). Each crew drew a numbered paper (1 = intervention, 2 = control) from a box allocating the crew to either intervention or control. Randomisation procedure was conducted under supervision of two researchers to ensure allocation concealment. Participants were not informed whether they were allocated to the intervention or control arms of the study.

Due to the nature of the study (participants knowledge of receiving ventilation feedback), true blinding of participants and outcome assessors was not possible, nor was blinding of researchers. However, the primary outcome measure for the study was not disclosed to participants prior to, or upon completion of participation. Measures were taken to blind the primary outcome of the study by administering a physical activity questionnaire, providing chest compression feedback to all participants, and collecting the perceived effort of chest compressions. This was done solely to mask the outcome of the study

Real-time ventilation feedback was enabled for the intervention group and disabled for the control group, though ventilation quality data were recorded for both groups.

### Equipment and simulation set-up

A ZOLL X-series defibrillator, an intubated Laerdal ALS mannequin (Laerdal Medical, Stavanger, Norway) with the AccuVent sensor, a bag-valve-mask and electrode pads attached was placed in advance by investigators.

The chest compression dashboard displayed compression depth and rate numerics, a release indicator bar and a Perfusion Performance Indicator (Fig. 3). The defibrillator displayed real-time chest compression feedback for both the intervention and the control group, while real-time ventilation feedback was only visible to the intervention group.

The configurable ventilation targets were set to a tidal volume of 550 ± 50 ml and ventilation rate of 9 ± 1 bpm based on guideline recommendations. The chest compression targets were set to a depth of 5–6 cm and a rate of 100–120 compressions per minute. When data is recording, a blue light is activated on the sensor and real-time feedback is displayed on the X-series monitor/defibrillator on the ventilation feedback dashboard (Fig. 3).

### Procedure and data collection

Participants allocated to the intervention group participated in a brief training on the defibrillator with integrated real-time ventilation feedback. Participants allocated to the control group were reminded of guideline ventilation targets (10:1 ratio for continuous compressions, 500–600 ml per ventilation) and were also given the opportunity to practice manual ventilations without real-time feedback displayed. Both groups were instructed on use of real-time chest compression feedback technology and given an opportunity to practice prior to the scenario.

Following the short training session each crew was given an identical standardised introduction explaining that they were to respond to an OHCA scenario and take over chest compressions and ventilations on an intubated mannequin. One rescuer was instructed to provide continuous ventilations to the patient using a bag-valve-mask while the other participant was instructed to perform chest compressions according to ERC guidelines.

Both groups performed a simulated cardiac arrest scenario of eight minutes, rotating roles (compressor or ventilator) every two minutes. The sequence of compression performance and ventilation delivery were recorded for each participant. At the end of each two-minute interval during the scenario, rescuers were asked to self-report their levels of perceived fatigue based on the modified Borg scale.^16^

Upon completion of the scenario, a member of the research team transferred data from the defibrillator to a secure server accessible to the study personnel only. The data file contained all information acquired from the defibrillator. No identifying information about the participants was included in the data files.

### Outcomes

The primary outcomes were ventilation quality, defined as ventilation rate (8–10 bpm) and tidal volume (500−600 ml) for a given breath, and ventilation rate and tidal volume as individual measurements.

### Sample size determination

With a sample size of 60 individuals (30 crews), the multiple linear regression test of ρ² = 0 (α = 0.05) for 6 normally distributed covariates would have 80% power to detect a ρ² of 0.203.

### Statistical methods

Test for normal distribution was conducted using the Shapiro–Wilk test. For the primary outcome (ventilation rate, tidal volume, ventilation quality) the Shapiro–Wilk test indicated that the data was not normally distributed (p < 0.001). The same tendency was found for numeric covariates (age, EMS experience, hand size, p < 0.001).

Primary outcome data were compared between intervention and control groups using the Wilcoxon rank-sum test. To determine covariance balance across the groups, a chi–square test was utilized for categorical variables while Wilcoxon rank-sum test was used for numeric variables.

Linear regression modelling was used to predict ventilation quality including participant covariates, consisting of intervention group, age, hand size, EMS experience, level of training, and cardiopulmonary arrest exposure in the past three years. A linear mixed model was also generated that included the six participant covariates with the introduction of the variable crew as a random effect. Statistical analysis was performed using SAS version 9.4.

## Ethics

We applied for ethical approval from The Danish National Committee on Health Research Ethics (journal number: H-19027602, 24 May 2019), but formal approval was waived. The study is reported according to the Consolidated Standards Of Reporting Trials (CONSORT) 2010 extensions for health care simulation research.^17^

## Results

### Participant characteristics

Of 34 crews (68 individuals) screened for inclusion, 2 crews (4 individuals) were excluded during this initial screening: 1 crew for physical limitations and 1 crew for being active first aid instructors (Fig. 1). Exclusion of one individual resulted in exclusion of the crew. Of the 32 crews (64 individuals) included in the study (55 males, 9 female), 16 crews (32 individuals) were allocated in the intervention group (real-time ventilation and chest compression feedback) and 16 crews (32 individuals) in the control group (real-time chest compression feedback only). No crews were excluded after randomisation (Fig. 1). There were no significant differences in baseline characteristics between participants in the intervention and control groups (Table 1).

**Table 1:** Participant baseline characteristics.

Variable	No ventilation feedback (n = 32)	Real-time ventilation feedback (n = 32)	Overall (N = 64)	P value
Age, Median (IQR)	35.5 (29.0–47.0)	32.5 (28.5–41.0)	33.5 (29.0–43.0)	0.371
Gender, n (%)				0.072
Male	30 (93.8)	25 (78.1)	55 (85.9)	
Female	2 (6.2)	7 (21.9)	9 (14.1)	
Level of training, n (%)				0.450
BLS	27 (84.4)	29 (90.6)	56 (87.5)	
ALS	5 (15.6)	3 (9.4)	9 (12.5)	
Years in EMS, median (IQR)	11.0 (5.3–21.5)	7.5 (3.8–15.0)	8.5 (4.8–17.0)	0.116
OHCA exposure, last 3 Yrs, N (%)				0.262
0 to 5	2 (6.2)	7 (21.9)	9 (14.1)	
5 to 10	11 (34.4)	12 (37.5)	23 (35.9)	
10 to 15	11 (34.4)	7 (21.9)	18 (28.1)	
15 or more	8 (25.0)	6 (18.7)	14 (21.9)	
Hand size in cm, Median (IQR)	20 (19–20)	19 (18–19)	19 (18–20)	0.058
Dominant hand, n (%)				0.491
Right	28 (87.5)	26 (81.3)	54 (84.4)	
Left	4 (12.5)	6 (18.7)	10 (15.6)	

IQR = Interquartile Range, BLS = Basic Life Support, ALS = Advanced Life Support, EMS = Emergency Medical Services, OHCA = Out-of-hospital cardiac arrest.

### Primary outcomes

Ventilation quality (percentage of guideline-compliant ventilations provided for both rate and volume) was significantly higher in the intervention group compared to the control group (p < 0.001, Table 2). The intervention group performed ventilations in adherence with guideline recommendations for 75.3% (interquartile range (IQR) 66.2%–82.9%) of delivered ventilations, compared to 22.1% (IQR 0%–44.0%) of ventilations provided by the control group (p < 0.001).

**Table 2:** Ventilation quality parameters.

Variable	No ventilation feedback (n = 16)	Real-time ventilation feedback (n = 16)	*P*
Ventilations in target rate (%)	66.7 (40.9–77.9)	97.4 (97.1–100)	< 0.001
Ventilations in target volume (%)	53.4 (8.4–66.7)	77.5 (64.9–83.8)	< 0.001
Ventilations in target rate and volume (%)	22.1 (0–44.0)	75.3 (66.2–82.9)	< 0.001

Data presented as median (Interquartile range).

N refers to the number of crews consisting of 2 providers.

**Fig. 4:**
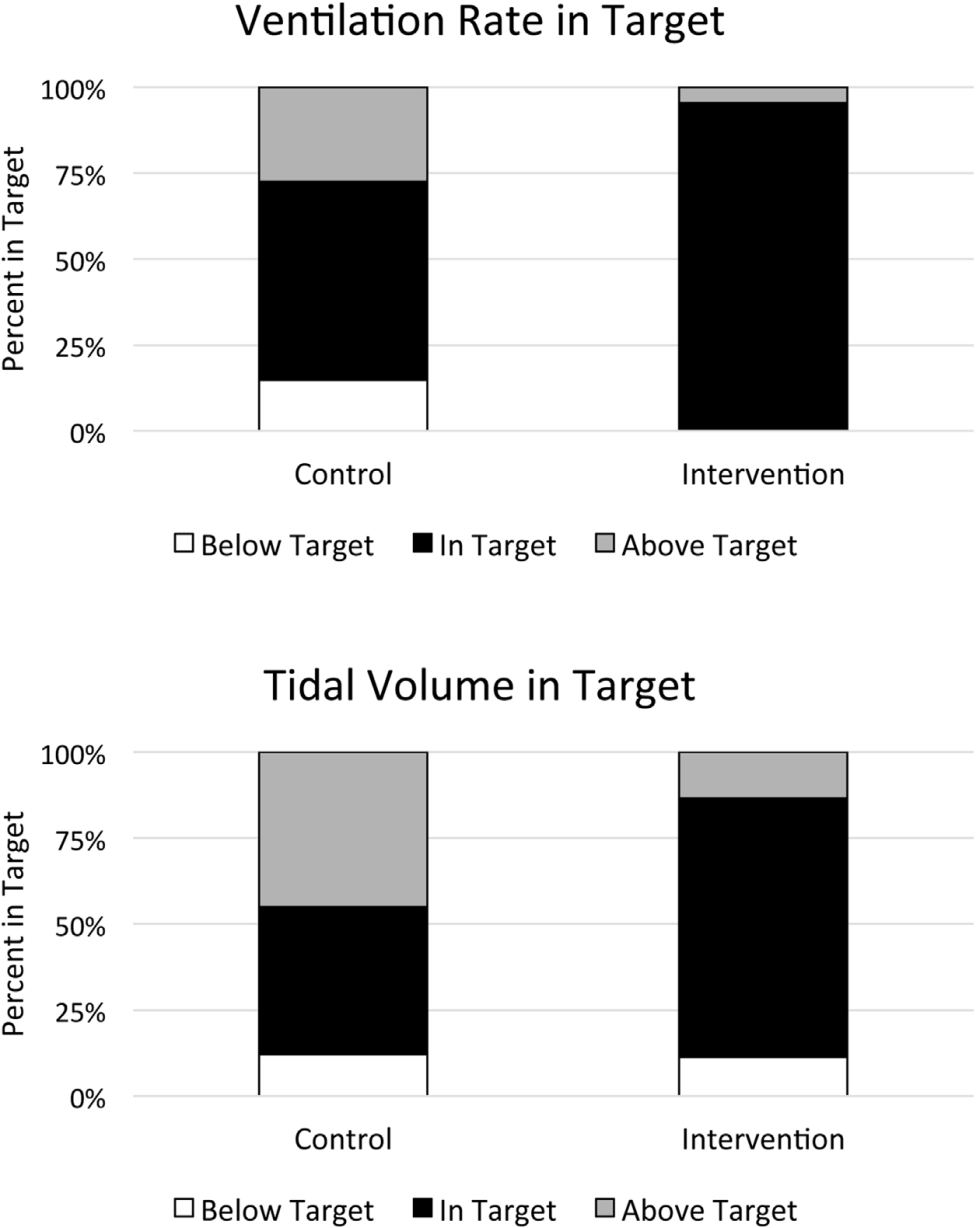
Percent in target for ventilation parameters.

The intervention group performed guideline-compliant ventilation rate during 97.4% (IQR 97.1%–100%) of ventilations, versus 66.7% (IQR 40.9%–77.9%) for the control group (p < 0.001). Tidal volume compliance was also higher in the intervention group, with 77.5% (IQR 64.9%–83.8%) of ventilations within target compared to 53.4% (IQR 8.4%–66.7%) delivered by the control group (p < 0.001) (Fig. 4). Additionally, there were no differences in chest compression quality metrics between ventilation feedback groups (Table 3).

**Table 3:** Chest compression quality parameters.

Variable	No ventilation feedback (n = 16)	Real-time ventilation feedback (n = 16)	*P*
Compressions in target rate (%)	96.6 (88.2–99.3)	93.6 (84.0–97.8)	0.214
Compressions in target depth (%)	74.2 (70.1–86.2)	77.4 (69.8–86.2)	0.791
Compressions in target rate and depth (%)	70.5 (64.6–80.9)	69.9 (56.0–82.7)	0.851

Data presented as median (Interquartile range).

N refers to the number of crews consisting of 2 providers.

When controlling for participant covariates (age, hand size, EMS experience, training level, and cardiac arrest exposure in the last three years) (multivariate model 1) of the intervention group, adherence to guidelines was 45.2% (0.452) [95% CI 0.352–0.551] higher in participants receiving ventilation feedback (p < 0.0001). Due to randomisation of participants by crew, the crew variable was introduced to the linear regression model as a random effect to generate a linear mixed model (multivariate model 2) using the PROC MIXED procedure in SAS. The mixed model revealed a 44.7% (0.447) [95% CI 0.326–0.567] higher guideline adherence with ventilation feedback when including crew as a random effect (p < 0.0001).

## Discussion

This randomised controlled trial investigated the use of real-time ventilation feedback during simulated OHCA resuscitation. Ventilation quality was superior when real-time ventilation feedback was used to guide ventilation, as compared to standard ventilation without ventilation feedback. These results were consistent when controlling for participant covariates and considering the randomisation protocol by crew.

High-quality CPR consists of guideline-compliant chest compression depth, rate and fraction, as well as ventilation rate and volume.^3^ The emergence of CPR feedback technology has primarily focused resuscitation efforts on the delivery of high-quality chest compressions, while less attention has been given to ventilation quality. Ventilation rate guidance has previously been incorporated into some monitor/defibrillators; however, there has been limited or no ability to change the ventilation rate target, determine tidal volume, or record ventilation data continuously. Transthoracic impedance-based breath detection and capnography have been used to determine real-time ventilation rate, though these technologies are subject to limitation and have been reported to overestimate ventilation rate. Furthermore, the reduced end-tidal carbon dioxide levels observed during hyperventilation could limit the prognostic utility of capnography during cardiac arrest.^18^, ^19^

In this study, real-time ventilation feedback was provided as an intervention during manual ventilation delivery to an intubated mannequin. The intervention group recorded ventilation quality performance of 75.3%, compared to only 22.1% for providers without the use of ventilation feedback. Given the heterogeneity among paramedics, regression modelling was used to control for differences in several factors that included age, hand size, cumulative and recent resuscitation experience, and level of training. Notably, we found a performance difference of 45.2% in ventilation quality when adjusting for these characteristics, indicating that real-time ventilation feedback may improve rescuer guideline adherence regardless of individual experience or training.

The observed improvement in rate may also have been achieved by applying a simple metronome (visual or audio) whereas the improvement in volume would not be possible as this requires the use of imputed feedback in contrast to autonomous. It is unclear if the gamification of the dashboard contributes to the ventilation quality.

Routine intubation for OHCA is debated and the intervention is regarded as secondary to BLS.^3^ As hyperventilation is reported in the majority of literature to compromise intrathoracic pressure and haemodynamics, airway management in OHCA may not only be regarded as a matter of securing airway patency, but also an opportunity to ensure high quality ventilation delivery. The clinical need for ventilation guidance is well-established by literature documenting the difficulty meeting guideline recommendations and the propensity for hyperventilation, regardless of the provider or the setting.^5^, ^6^, ^7^, ^20^, ^21^

In our study, efforts were made to replicate OHCA by the setup and the introduction given to participants to allow for more reliable interpretation of the findings transferred to a clinical context. However, the evidence on transfer of medical skills from simulation to clinical setting is sparse. A review by Okuda et al. found that only a few studies showed improved patient outcome from simulation.^22^ Therefore, and as this is an early stage study in a simulation environment, no claims can be made regarding sustainability of changes in performance, or outcomes in the clinical setting.

Introducing a ventilation feedback dashboard in addition to the CPR feedback dashboard could potentially lead to decreased compression performance due to information overload. This did not seem to be the case (Table 3), as there was no significant difference in chest compression performance between groups. However, all crews were accustomed to the use of feedback for chest compressions.

Monitoring ventilation quality is vital to other non-cardiac arrest patients as well. In patients with traumatic brain injury (TBI) it is imperative to prevent secondary insults due to hypoxia, hypocapnia, and hypotension.^23^ Pre-hospital provider adherence to TBI guidelines is associated with increased odds of survival in patients with severe injury, emphasizing the importance of proper ventilation in this specific population.^23^ The findings of our study may therefore be useful for other patient populations besides OHCA.

The use of ventilation feedback systems may aid clinicians in delivering appropriate ventilation during cardiopulmonary arrest. This novel real-time ventilation feedback technology might also be implemented in tandem with other technologies, such as end-tidal carbon dioxide or chest compression feedback. Furthermore, the use of these devices also allows for the recording of data over a resuscitation to be used for debriefing, quality assurance, or research purposes.

### Limitations

This study is limited in that the data are reflective of provider performance during simulated conditions of cardiopulmonary arrest, thus the results should be interpreted in this context. Further study in a clinical setting is warranted to determine the effects of real-time ventilation feedback on rescuer performance and patient outcomes.

In our study the monitor was pre-positioned. In a clinical setting positioning the monitor correctly is vital to achieve effect of the feedback why the pre-positioning of the monitor serves as a limitation.

Data were collected using an intubated mannequin. Use of the Accuvent device is not limited to intubated patients and may be placed in the airway between the bag and the mask or SGA. It is essential that the provider maintains a tight seal on the mask to ensure the tidal volume measured by the device is delivered to the patient. It is, however, possible that the seal may be suboptimal where a facemask or SGA is deployed, an issue requiring further study.

Despite efforts to mask the purpose of the study, we cannot rule out that participants may have changed their regular ventilation performance or that the results may be subject to the Hawthorn effect.^24^

## Conclusion

Paramedics provided real-time ventilation feedback achieved higher guideline compliance with both ventilation rate and tidal volume during manual ventilation compared to paramedics that received no real-time ventilation feedback. Providing real-time feedback during resuscitation has the potential to improve ventilation quality and may allow providers to avoid harmful hyperventilation of OHCA patients. Further research examining the feasibility and impact on patient outcomes using real-time ventilation feedback in a clinical setting is warranted.

## Funding

The study is funded by ZOLL Medical Corporation, Chelmsford, MA and conducted by Copenhagen Emergency Medical Services under the Danish “work for hire” legislation. Funders participated in designing the study, data collection, statistical analysis and writing the manuscript.

## Conflict of interest

L. Clark and A. Silver are employees of ZOLL Medical Corporation, which manufactures the devices that were used in this investigation. Both contributed to study design, analysis and reporting. They approved the final manuscript but the final decision to publish was made by all authors.

This study was conducted by Copenhagen EMS under the work for hire legislation from which Copenhagen EMS received funding from ZOLL Medical Corporation for expenses related to this project.

## CRediT authorship contribution statement

**Rasmus Meyer Lyngby:** Project administration, Conceptualization, Investigation, Methodology, Formal analysis, Data curation, Writing - original draft. **Lyra Clark:** Resources, Software, Methodology, Writing - review & editing. **Julie Samsoee Kjoelbye:** Data curation, Validation, Supervision, Writing - review & editing. **Roselil Maria Oelrich:** Data curation, Validation, Writing - review & editing. **Annemarie Silver:** Resources, Software, Methodology, Writing - review & editing. **Helle Collatz Christensen:** Methodology, Formal analysis, Supervision, Writing - review & editing. **Charlotte Barfod:** Project administration, Resources, Supervision, Writing - review & editing. **Freddy Lippert:** Conceptualization, Funding acquisition, Project administration, Methodology, Writing - review & editing. **Dimitra Nikoletou:** Methodology, Supervision, Writing - review & editing. **Tom Quinn:** Conceptualization, Methodology, Supervision, Formal analysis, Writing - review & editing. **Fredrik Folke:** Conceptualization, Funding acquisition, Project administration, Supervision, Formal analysis, Methodology, Writing - review & editing.
