# Supplementary material for: Higher resuscitation guideline adherence in paramedics with use of real-time ventilation feedback during simulated out-of-hospital cardiac arrest: A randomised controlled trial
Source: Resusc Plus. 2021 Jan 30;5:100082. doi: 10.1016/j.resplu.2021.100082 (PMC8244327; doi:10.1016/j.resplu.2021.100082)
Supplement: Supplementary file 2 [file mmc2.pdf]

Manual Ventilation Quality during CPR with and without use of Real-time Feedback Technology

Study Number - 34701
Version: 1.0
February 25, 2019

269 Mill Road
Chelmsford, MA 01824-4105

Clinical Study Protocol

CONFIDENTIAL
This confidential document is the property of ZOLL Medical Corporation. No unpublished information contained herein may be disclosed without the prior written approval of ZOLL Medical Corporation. This study will be conducted in accordance with ISO14155. Essential documents will be archived by the sponsor. Dissemination may only be made with the express written permission of ZOLL Medical Corporation.

Sponsor

**Sponsor:**	ZOLL Medical
**Primary Contact:**	David Appleby
**Address:**	269 Mill Road<br>Chelmsford, MA 08124-4105
**Phone Number:**<br>**Email:**	978-421-9190<br>dappleby@zoll.com

Principle Investigator

**Investigator:**	Freddy Lippert, MD
**Address:**	Copenhagen Emergency Medical Services<br>Telegrafvej 5<br>DK-2750 Ballerup
**Phone Number:**<br>**Email:**	+45 38 69 80 00<br>freddy.lippert@regionh.dk

List of Abbreviations

AE	Adverse Event
CPR	Cardiopulmonary Resuscitation
CRF	Case Report Form
eCRF	Electronic Case Report Form
EMS	Emergency Medical Services
FDA	Food and Drug Administration
GCP	Good Clinical Practice
HIPAA	Health Insurance Portability and Accountability Act
IB	Investigator’s Brochure
IRB	Institutional Review Board
PHI	Protected Health Information
PI	Principal Investigator
SAE	Serious Adverse Event
SOP	Standard Operating Procedure

Table of Contents

1 *Introduction* 7
1.1 Background 7
1.2 Risks/Benefits 8
1.2.1 Risks 8
1.2.2 Benefits 8
2 Study Protocol 8
2.1 Study Design 8
2.1.1 Purpose 9
2.1.2 Scope and Duration 9
2.1.3 Sample Size 9
2.2 Study Objectives 9
2.2.1 Primary Outcome Measure 9
2.2.2 Secondary Outcome Measures 9
2.3 Subject Screening and Eligibility Criteria 9
2.3.1 Subject Recruitment and Screening 9
2.4 Study Procedures 10
2.4.1 Subject Introduction and orientation 10
2.4.2 Training 11
2.4.3 Cardiac Arrest Scenario 11
2.4.4 Equipment 12
2.4.5 Subject Enrollment 12
2.4.6 Case Report Forms and Evaluations 13
2.5 Classification of Adverse Events and Reporting of Adverse Events 14
2.5.1 Adverse Event Reporting 15
2.6 Subject Withdrawal 15
2.7 Protocol Deviations 15
3 Statistical Analysis 15
3.1 Randomization 15
3.2 Blinding 16
3.3 Sample Size 16
4 Monitoring Procedures 16
4.1 Monitoring 16
4.2 Investigator/Site Selection 16
4.3 Case Report Form Review 16
4.4 Source Documents 17
4.5 Interim On-site Monitoring 17
4.6 Discharge Monitoring Visit 17
5 Study Administration 17
5.1 Study Sponsor 17
5.2 Applicable Regulations 18
5.2.1 Ethics Committee 18

5.2.2 Informed Consent....18
5.3 Records and Reports....18
5.3.1 Investigator Records....18
5.3.2 Sponsor Records....19
5.4 Publications....19
5.5 Study Discontinuation....20
5.6 Use of Study Findings....20
5.6.1 Subject Confidentiality....20
5.6.2 Ownership of Study Data....21
6 References....22

Protocol Summary

**Title**	**Manual Ventilation Quality during CPR with and without use of Real-time Feedback Technology**
**Short Title**	Ventilation Quality Feedback
**Study Design**	Prospective, randomized controlled study
**Study Duration**	3 months
**Study Site(s)**	Copenhagen Emergency Medical Services, Copenhagen, Denmark
**Objective(s)**	To determine if real-time visual ventilation feedback improves ventilation quality during simulated cardiac arrest.
**Primary Analysis**	Multiple linear regression
**Number of Subjects**	60
**Subject Eligibility Criteria**	**Inclusion Criteria**<br>Candidates for this study must meet all of the following Inclusion criteria:<br>1. Advanced Life Support or basic life support-certified Emergency Medical Services personnel with current CPR certification<br>2. Perform CPR as a job requirement<br>3. Exposure to minimum of 1 cardiac arrest patient case or completion of CPR training in the previous 6 months<br>4. Age ≥ 18 years<br><br>**Exclusion Criteria**<br>Candidates will be excluded from the study if any of the following conditions are present:<br>1. CPR instructor<br>2. Known back, upper extremity (hand, elbow or shoulder), cardiac, or other health problems that limit physical job performance<br>3. Known pregnancy

1 *Introduction*

1.1 *Background*

Over 350,000 people experience sudden out-of-hospital cardiac arrest (OHCA) in the United States each year and less than 15% of those treated by EMS personnel survive to hospital discharge[1]. Sudden cardiac arrest is a life-threatening condition resulting from the cessation of cardiac function and the subsequent loss of blood circulation. Cardiopulmonary Resuscitation (CPR) is a procedure used by rescuers that comprises chest compressions and rescue breathing in an effort to restore spontaneous circulation. In addition to high quality chest compressions, ventilations must be delivered to the patient at an appropriate rate and volume in order to optimize flow of oxygenated blood to vital organs.

The European Resuscitation Council currently recommends delivering 1 breath every 6 seconds (10 breaths per minute) while continuous chest compressions are being performed on patients with secured airways[2]. Recommendations state to avoid hyperventilation (both excessive rate and tidal volume). However, hyperventilation is a frequently reported occurrence during the treatment of OHCA patients[3,6,8]. A clinical observational study by Aufderheide and colleagues reported 7 cases where patients were ventilated at a mean rate of 37 breaths per minute (bpm) [3]. Despite all EMS personnel being re-trained during the study to deliver 12 bpm, the remaining 6 patients in the study received a mean rate of 22 bpm[3]. Furthermore, the reduction in rate from 37 to 22 bpm resulted in longer ventilation times, such that percent times of positive airway pressure were similar between the two groups of patients. Hyperventilation has also been reported in the hospital setting[4,6]. Milander et al. observed twelve in-hospital cardiac arrests during which patients were ventilated by respiratory therapists at a mean rate of 37 bpm (range: 24 - 60 bpm)[4].

Pre-clinical studies have highlighted the detrimental effect of hyperventilation on hemodynamics during cardiac arrest. Myocardial perfusion, cardiac output, and blood flow to the brain have all been shown to decline with hyperventilation[3,5], and a separate study reported that cerebral and coronary perfusion pressures significantly increase when ventilation rates are reduced[7].

While frequent reports of healthcare providers performing CPR outside of guidelines can be found in the literature, it has been demonstrated that chest compression quality can be improved by providing guidance. In a laboratory study, 41 participants performed CPR on pigs with and without guidance from a metronome. Initial baseline compressions without guidance were below guidelines for rate (72 cpm), while all participants were able to match 100 cpm with the metronome guidance turned on[4]. Similarly, use of real-time feedback has been shown to significantly improve chest compression performance[9], and can lead to improved patient outcomes[10]. In contrast to compression quality, there is little

information on the effect of feedback on ventilation performance. We hypothesize that the use of real-time visual feedback during CPR will improve manual ventilation quality.

1.2 *Risks/Benefits*

1.2.1 Risks

Potential risks of participating in this study include: 1) stress or anxiety in answering a questionnaire or by being observed while performing a task, and 2) the physical stress of performing CPR-related skills. For the healthcare providers, the physical risks associated with performing CPR in this study are no more than those encountered during the normal performance of CPR in their daily duties as an EMS provider. The physical risks may include physical exertion resulting in fatigue or musculoskeletal (back, shoulder, arm or hand) discomfort. Rare instances have been published in which individuals with undiagnosed but underlying heart disease have suffered a heart attack or cardiac arrest (death) while performing CPR.

The risks will be minimized by only selecting previously trained participants who are not limited by back or upper extremity injury/pain, cardiac history, or other health problems. The study will be conducted at a facility where immediate access to trained rescuers, emergency care and defibrillation is available.

1.2.2 Benefits

Participants may benefit from practicing and training with ventilation feedback.

2 Study Protocol

2.1 *Study Design*

The proposed CPR quality project will be conducted at Emergency Medical Services Copenhagen, Denmark in collaboration with ZOLL Medical Corp. The study is designed to assess the effect of visual feedback on the quality of manual ventilations provided by BLS and ALS trained rescuers during a simulated cardiac arrest scenario. Ventilation quality will be quantified by rate and volume. Rescuers will be randomized to perform CPR either with or without real-time ventilation feedback. Rescuers randomized to the feedback group will participate in a brief training on the device’s integrated real-time ventilation feedback and a brief practice session with the technology. Rescuers randomized to the control group without feedback will be reminded of the Guidelines ventilation targets and will be

given the opportunity to practice manual ventilations prior to the scenario. Both groups will then perform the simulated cardiac arrest scenario in order to assess the effect of ventilation feedback on performance.

2.1.1 Purpose

The purpose of this study is to determine if real-time visual ventilation feedback improves manual ventilation quality during CPR in a simulated cardiac arrest scenario.

2.1.2 Scope and Duration

This is a prospective, randomized controlled study that will be conducted for approximately 3 months.

2.1.3 Sample Size

60 subjects will be enrolled in this study. With a sample size of 60, the multiple regression test of $\rho^2=0$ ($\alpha = 0.05$) for 6 normally distributed covariates will have 80% power to detect a ρ^2 of 0.203.

2.2 *Study Objectives*

To determine if real-time visual ventilation feedback improves ventilation quality during simulated cardiac arrest.

2.2.1 Primary Outcome Measure

The primary outcome measure for the study is ventilation quality (percent in target range). Ventilation rate (breaths * min^{-1}) and tidal volume (ml) will be measured to assess ventilation performance.

2.2.2 Secondary Outcome Measures

Secondary outcome measures will be assessment of the effects of participant characteristics, such as hand size, dominant hand, and ventilation technique, on the quality of manual ventilations. Participant's length of job experience in performing CPR will also be measured.
An additional secondary outcome measure is rating of perceived effort while performing CPR. Participants will be asked to rate levels of perceived effort at the end of every two-minute interval during the protocol (Modified Borg Scale).

2.3 *Subject Screening and Eligibility Criteria*

2.3.1 Subject Recruitment and Screening

Employees who are BLS or ALS certified and meet the following inclusion and exclusion criteria will be recruited to participate verbally or via email.

2.3.1.1 Inclusion Criteria

Candidates for this study must meet all of the following Inclusion criteria:

1. Advanced Life Support or basic life support-certified Emergency Medical Services personnel with current CPR certification
2. Perform CPR as a job requirement
3. Exposure to minimum of 1 cardiac arrest patient case or completion of CPR training in the previous 6 months
4. Age $\geq$ 18 years

2.3.1.2 Exclusion Criteria

Candidates will be excluded from the study if any of the following conditions are present:

1. CPR instructor
2. Known back, upper extremity (hand, elbow or shoulder), cardiac, or other health problems that limit their physical job performance
3. Known pregnancy

2.4 *Study Procedures*

2.4.1 Subject Introduction and orientation

Participants will be grouped into pairs for the study. After a brief standardized introduction and orientation, participants will read and sign informed consent forms. This will be followed by an introduction to the manikin, equipment used in the study, and the simulation protocol in order to orient participants. Participants will also be asked to complete three short surveys:

- ***Demographic survey***
  - Age
  - Gender
  - Height
  - Weight
  - Hand size
  - Dominant hand
  - Level of training: ALS/BLS
  - Number of years in EMS

Estimated number of cardiac arrest case exposure in previous 3 years (0-5, 5-10, 10-15, 15+)

- ***International Physical Activity Questionnaire***
  - A brief 4-question survey to determine weekly physical activity

- ***Situational Fatigue Scale***
  - A 13-question Likert scale survey to assess trait fatigue as fatigue is known to affect CPR performance[11,13].

2.4.2 Training

Following completion of the surveys, the subjects will be randomized to the ventilation feedback group, or a group receiving no ventilation feedback. The subjects assigned to the ventilation feedback group will be introduced to the feature on the monitor/defibrillator that provides real-time ventilation feedback and participate in a brief training session. The subjects who will not receive ventilation feedback will be reminded of the Guidelines for ventilation and practice manual ventilations for a brief period. Once training and practice have been completed, both groups will then be asked to respond to a cardiac arrest scenario.

2.4.3 Cardiac Arrest Scenario

The manikin will be intubated with a bag-valve-mask attached and electrode pads will be placed on the manikin in advance by investigators. The X series monitor will be powered on in manual mode.

A team of two participants will be instructed to respond to a simulated cardiac arrest using a monitor in standard manual mode. One participant will continuously ventilate the patient using a bag-valve-mask while the other participant will perform chest compressions. The clinicians will follow their standard clinical practices (i.e. ERC guidelines for continuous compressions/ventilations). The participants will trade roles after two minutes of CPR as instructed by a study investigator. This cycle will be repeated such that each participant performs 4 minutes of ventilations and 4 minutes of chest compressions. The scenario should last 8 minutes. The sequence of compression performance and ventilation delivery will be recorded for each participant to account for time differences during the scenario. At the end of each two-minute interval during the scenario, rescuers will be asked to self-report their levels of perceived fatigue based on the modified Borg scale[12].

The participants will perform CPR with their hands placed on a chest compression measuring device (CPR sensor) positioned on the manikin's

chest. The CPR sensor, which is integrated into commercially available ZOLL defibrillator electrodes, contains an accelerometer that senses chest compressions and measures the depth, rate, and timing of chest compressions. Data from the accelerometer are recorded in the X series monitor and can be downloaded and viewed after the event in Code Review (ZOLL Data Systems; Broomfield, CO) or Case Review (ZOLL Data Systems).

Real-time ventilation data will be displayed for the group randomized to ventilation feedback (during Training session and Scenario). Ventilation data will be recorded for both groups using a monitor/defibrillator (ZOLL X Series, ZOLL Medical Corp., Chelmsford, MA) for the duration of the protocol.

2.4.4 Equipment

The project requires use of 1) ZOLL X series monitors/defibrillator equipped with real-time ventilation feedback technology, 2) disposable flow sensors with reusable cables that attach to the X Series, 3) bag-valve-masks, 4) CPR pads containing the accelerometer and 5) manikins. Data collected on the X Series will be transferred to a PC via a USB flash drive and stored for off-line analysis. All equipment will be provided at no cost to EMS CPH to be used for the purposes of the proposed project.

2.4.4.1 Ventilation Feedback

- **Ventilation Volume Numeric-** Displays the absolute inspiratory volume. The numeric is green if the value is within 75 mL of the target volume of 550 mL and yellow when outside of the target range.
- **Ventilation Rate Numeric-** Displays the ventilation rate. The numeric is green if the value is 8-10 bpm and yellow if outside of the target range.
- **Ventilation Quality Indicator** - to achieve a target volume of 550 mL and rate of 8-10 bpm through visual feedback. Participants will attempt to fill a circle on the monitor with each ventilation. If a ventilation is performed at 550 ± 75 mL and a rate of 8-10 bpm, the circle will remain green. If a ventilation is performed outside of that range, the circle will turn yellow. A countdown timer is displayed within the indicator to prompt the user to ventilate at the target of 9 bpm.

2.4.5 Subject Enrollment

Subjects are enrolled in this study when they have:

- Met all eligibility criteria
- Agree to participate by signing an informed consent form

Evaluation Requirements

2.4.5.1 *Assessments*

The following data points will be recorded:

- *Demographic Characteristics*
  - Age
  - Gender
  - Height
  - Weight
  - Hand size
  - Dominant hand
  - Level of training: ALS/BLS
  - Estimated number of cardiac arrest case exposure in previous 3 years (0-5, 5-10, 10-15, 15+)
  - Number of years in EMS

- *International Physical Activity Questionnaire*
  - A brief 4-question survey to determine weekly physical activity.

- *Situational Fatigue Scale*
  - A 13-question Likert scale survey to assess trait fatigue as fatigue is known to affect CPR performance[11,13].

2.4.6 Case Report Forms and Evaluations

The electronic case report form (eCRF) is the primary data collection instrument for the study. All data requested on the eCRF must be recorded. All missing data and errors must be explained by edit checks.

If a correction is required for an eCRF, the time and date stamps track the person entering or updating eCRF data and creates an electronic audit trail to reflect the reason for the change.

If the electronic data cannot be directly entered onto the eCRFs, these data should be created, imported, stored and maintained appropriately to ensure data and system security.

A member of the research team will transfer data from the X Series defibrillator to a PC and upload to a secure server that is password protected and can be accessed only by study personnel. The data file will contain all information acquired from the defibrillator including ECG (generated by a simulator), defibrillator settings, chest compression quality information,

ventilation quality information, etc. No identifying information about the participants will be included in the data files.

2.4.6.1 *Eligibility Evaluation CRFs*

The Eligibility Evaluation CRF includes the Inclusion/Exclusion Checklist.

Inclusion/Exclusion Checklist

The investigator or designee will review the inclusion and exclusion criteria. This form includes the inclusion and exclusion criteria checklist or validation.

2.4.6.2 *Protocol Deviation CRF*

This form will be used to document any protocol deviations in the study. Please see Section 2.7 and Protocol Deviation CRF for additional information regarding Protocol Deviations.

2.4.6.3 *Subject Discontinuation CRF*

This form will be used to document a subject's reason for exiting the study. Please see Section 2.6 for additional information regarding Subject Discontinuation.

2.4.6.4 *Adverse Event CRF*

All adverse events and complications will be recorded and reported on the Adverse Event CRF. Serious adverse events must be reported to the Sponsor within 24 hours, but no later than 10 days after made aware of the event.

> We do not anticipate any Adverse Events (AE) for this study beyond what would be expected during typical professional duties of our participants including potential electrical shock from the defibrillator, and the ability to perform CPR despite its potential strenuous physical activity. Any AEs resulting from involvement in the medical simulation, use of the defibrillator or feedback device or in delivering CPR at any of the study sites shall be reported to the study PI.

2.5 *Classification of Adverse Events and Reporting of Adverse Events*

Serious adverse events must be reported to the Sponsor within 24 hours of becoming aware of the event, but no later than 10 days after the event. The Investigator is responsible for reporting adverse events to their individual IRBs.

Serious adverse event is defined as:

- Death

- A serious deterioration in the health of the patient that results in a life-threatening illness or injury
- A permanent impairment of a body structure of body function
- In-patient hospitalization or prolongation of existing hospitalization
- Medical or surgical intervention to prevent permanent impairment to body structure or function
- Fetal distress, fetal death or congenital abnormality or birth defect.

2.5.1 Adverse Event Reporting

Adverse event reporting will comply with the regulations of the local IRB.

2.6 *Subject Withdrawal*

An enrolled subject is considered 'withdrawn' from the study if their participation in the study is discontinued for any reason after enrollment. Possible reasons for withdrawal include:

- Eligibility Criteria not met
- Investigator judgment

If a subject withdraws from the study, a Subject Discontinuation CRF will be completed.

2.7 *Protocol Deviations*

An Investigator shall notify the sponsor and reviewing IRB of any deviations from the study protocol that are done to protect the life or physical well-being of a subject. Such notice shall be given as soon as possible, but no later than 5 working days after the deviation occurred.

A protocol deviation is defined as a deviation from the study protocol.

Protocol deviations will be documented on a Protocol Deviation CRF.

3 Statistical Analysis

3.1 *Randomization*

Participants will be randomized to perform cardiopulmonary resuscitation either with or without real-time ventilation feedback. Rescuers randomized to the feedback group will participate in a brief training on the device's integrated real-time ventilation feedback. Both groups will then perform the simulated cardiac

arrest scenario in order to assess the effect of ventilation feedback on performance.

3.2 *Blinding*

Participants will not be blinded due to the knowledge of receiving ventilation feedback.

3.3 *Sample Size*

60 participants will be enrolled in this study. With a sample size of 60, the multiple linear regression test of $\rho^2=0$ ($\alpha = 0.05$) for 6 normally distributed covariates will have 80% power to detect an ρ^2 of 0.203.

4 Monitoring Procedures

4.1 *Monitoring*

This study will be monitored according to appropriate regulations and SOPs. The Investigator will allocate adequate time for such monitoring activities. The Investigator will also ensure that the monitor is given access to all the study-related documents and related facilities, and has adequate space to conduct the monitoring visit.

4.2 *Investigator/Site Selection*

The following documents must be received prior to site initiation and maintained at the site and Sponsor:

- Signed Clinical Study Agreement;
- Current Medical License;
- Current Curriculum Vitae (CV) for Primary Investigator(s) – dated and signed
- IRB/EC Approval Letter; and
- IRB Membership List and/or Accreditation Statement

Additional items that may be collected, are preferably collected prior to initiation, but not required at that time:

- CV's for Co-Investigator(s) – dated and signed; and
- Site Personnel Training Record(s).

4.3 *Case Report Form Review*

A database that is compliant with 21 CFR Part 11 will be used. eCRFs received from the site will be documented with their date of receipt and entered into the study database. If there is missing, incomplete, unclear or discrepant information recorded on eCRFs, a Data Clarification Form (DCF) will be generated. The DCF will be sent to the Investigator for completion and signed by the appropriate site personnel. The DCF will then be sent back to the

Sponsor to update the database. All common modules are demographics, screening, adverse events, labs, medical history, medications, procedures, treatment discontinuation, study completion, and signatures.

4.4 *Source Documents*

Source data is all information, original records of clinical findings, observations, or other activities in a clinical study necessary for the reconstruction and evaluation of the study. Examples of source data include: Patient Medical Records, recorded data from automated instruments, and subject files involved in the clinical study.

4.5 *Interim On-site Monitoring*

The monitor will maintain personal contact with the Investigator and staff by fax, phone, mail, and on-site visits. The monitor will require access to all subjects' records at each site visit. These may include:

- Investigator Administrative Records
- Subject Binders

Monitoring will verify:

- Protocol and regulatory compliance;
- Verification of data recorded on Case Report Forms to source documents; and
- Adequate subject enrollment.

4.6 *Discharge Monitoring Visit*

At the close of the study, the monitor will make a discharge on-site visit. The purpose of this visit is to:

- Collect all outstanding study data;
- Insure that the Investigator's files are accurate and complete;
- Review record retention requirements with the Investigator;
- Assure that all requirements are met for the closure of the study.

5 Study Administration

5.1 *Study Sponsor*

ZOLL Medical is the study sponsor and responsible for selecting qualified Investigators and providing them with the information they need to conduct the study properly, ensuring proper monitoring of the clinical site, ensuring that IRB review and approval are obtained, and ensuring that any reviewing IRBs are promptly informed of significant new information about the study.

5.2 *Applicable Regulations*

The following regulations were considered in developing/designing this study and apply to the ongoing conduct of the study:

- ISO 14155

5.2.1 Ethics Committee

Prior to participating in the study, the Investigator must forward written approval from the appropriate reviewing EC. The Investigator is responsible for obtaining and maintaining EC approval to participate in this study and forwarding evidence of EC notification of approval, disapproval, or study termination to Sponsor.

5.2.2 Informed Consent

Subjects must be willing and competent to sign informed consent at the time of enrollment in order to participate in the study.

5.3 *Records and Reports*

5.3.1 Investigator Records

The Investigator is responsible for the preparation (review and signature) and retention of the records cited below. Records are subject to inspection and must be retained for a period of at least two (2) years (or according to local regulatory requirements) after the study is terminated or the date that the records are no longer required for purposes of supporting publications.
Records to be maintained by the Investigator include:

- Study protocol and all amendments
- Signed Clinical Study Agreement
- IRB approval letter, including informed consent
- IRB membership list
- Correspondence relating to the study
- CVs for all Investigators
- Site personnel signature list
- Clinical monitor sign-in log
- Blank set of CRFs and instructions for completion
- Patient enrollment log
- Reports (includes annual reports, discharge reports from Investigator and Sponsor)

The following records must be maintained for each subject enrolled in the study:

- All data entered (e)CRF’s
- Supporting documentation of any complications

ZOLL reserves the right to secure data clarification and additional medical documentation on subjects enrolled in this study.

5.3.2 Sponsor Records

ZOLL Medical will maintain the following accurate, complete, and current records that are cited below.

5.3.2.1 *Sponsor Administrative Records*

- Enrolled Subject Identification Log
- Site Training and Monitoring Log
- Device Accountability Records
- Non-Disclosure Agreement
- Clinical Trial Agreement
- Investigator Information
    - Current CV(s)
    - Current Medical License
    - Financial Disclosure Forms(s)
- Monitoring
    - Monitoring Letter(s)
    - Monitoring Visit Report(s)
    - Site Visit Report(s)
    - Site Personnel Training Record(s)
- IRB
    - IRB roster or letter of assurance
    - IRB Approval Letter(s)
- All study conduct related correspondence with an IRB or FDA
- All study conduct correspondence with Site
- Required reports (reference Table 5)
- Documents Received Tracking Log
- Study protocol and previous revisions

5.3.2.2 *Subject Records*

- Original completed CRFs
- Copies of all completed logs and other subject specific forms (originals are maintained at site until the end of the study)
- Original subject specific protocol deviations
- Adverse event supporting data (e.g. EMS reports, medical records)
- Original completed Data Clarification Forms (DCFs)

5.4 *Publications*

In general, publications using study data will be managed as follows:

Authorship Selection

Authors will be selected based on the following criteria:

- Providing substantive contributions to the writing and development of the resulting publication; and either:
- Investigator's participation in the study design process; or
- Investigators participation in the study analysis process

Confidentiality

The Sponsor and authors will assure that no information, which would reveal a subject's identity, is used in any publication. Information, which could be used to establish a subject's identity, should not be provided to the Sponsor. Authors and the Sponsor will take every reasonable precaution to protect the identity of subjects enrolled in the study.

Review of Communication Guidelines

Investigators will receive a communication regarding the authorship selection, the publication co-authors and to which scientific platform the publication will be submitted.

5.5 *Study Discontinuation*

Sponsor will notify the Investigator of the completion or termination of the study or of the Investigator's participation in the study. The Sponsor will provide a summary to the reviewing IRB of the study within 6 months of study termination.

5.6 *Use of Study Findings*

5.6.1 Subject Confidentiality

In conducting the study, the investigational site will comply with all applicable laws and regulations relating to the confidentiality and security of individually identifiable medical information including but not limited to the requirements of the Health Insurance Portability and Accountability Act (HIPAA).

In the event that a subject revokes authorization to collect or use PHI, the Investigator, by regulation, retains the ability to use all information collected prior to the revocation of subject authorization. For subjects that have revoked authorization to collect or use PHI, attempts should be made to obtain permission to collect at least vital status (i.e. the subject is alive and well) at the end of their scheduled study period.

All personal information pertaining to subjects will be kept confidential. Subjects will be identified only by their Subject ID Number. Clinical study documents and hospital and clinic medical records pertaining to subjects may be reviewed by Sponsor personnel or their representatives.

The data will be stored on the defibrillator and will be transferred to a PC upon completion of the code scenario. The data files will then be uploaded to a secure server for later analysis by study personnel. All data will be secured in a password-protected computer or filed in a locked room. Files from this study will be kept for 10 years. Data will also be securely stored by ZOLL in Clindex.

Information obtained in the course of executing this study, including still and motion photography, may be presented for regulatory, clinical or educational purposes as long as no subject is identified.

5.6.2 Ownership of Study Data

The cumulative set of data collected by ZOLL Medical from the Investigator is considered ZOLL's data.

6 References

[1] Benjamin EJ, Virani SS, Callaway CW, Chamberlain AM, Chang AR, Cheng S, Chiuve SE, et al. (2018). Heart Disease and Stroke Statistics-2018 Update: A Report From the American Heart Association”. *Circulation*, 137(12), e67–e492

[2] Monsieurs KG, Nolan JP, Bossaert LL, Greif R, Maconochie IK, Nikolaou NI, Perkins GD, et al. (2015). European Resuscitation Council Guidelines for Resuscitation 2015. *Resuscitation*, 95,1-80.

[3] Aufderheide TP, Sigurdsson G, Pirrallo RG, Yannopoulos D, McKnite S, von Briesen C, Sparks CW, Conrad CJ, Provo TA, Lurie KG. (2004). Hyperventilation-Induced Hypotension During Cardiopulmonary Resuscitation. *Circulation*, 109(16), 1960–1965.

[4] Milander MM, Hiscok PS, Sanders AB, Kern KB, Berg RA and Ewy GA. (1995). Chest Compression and Ventilation Rates during Cardiopulmonary Resuscitation: The Effects of Audible Tone Guidance. *Academic Emergency Medicine*, 2: 708–713.

[5] Karlsson T, Stjernström E–L, Stjernström H, Norlén K and Wiklund L. (1994). Central and regional blood flow during hyperventilation. *Acta Anaesthesiologica Scandinavica*, 38: 180–186.

[6]O’Neill JF, Deakin CD. (2007). Do we hyperventilate cardiac arrest patients? *Resuscitation*. 73: 82-85

[7]Yannopoulos D, McKnite S, Tang W, Zook M, Roussos C, Aufderheide TP, Idris AH, Lurie KG. (2005) Reducing ventilation frequency during cardiopulmonary resuscitation in a porcine model of cardiac arrest. *Respiratory Care*, 50: 628-35.

[8] Aufderheide TP, Lurie KG. (2004). Death by hyperventilation: a common and life-threatening problem during cardiopulmonary resuscitation. *Critical Care Medicine,* 32: S345-51.

[9]Hostler D, Everson-Stewart S, Rea TD, Stiell IG, Callaway CW, Kudenchuk PJ, Sears GK, Emerson SS, Nichol G. (2011). Effect of real-time feedback during cardiopulmonary resuscitation outside hospital: prospective, cluster-randomised trial. *Bmj*. 342: d512.

[10]Bobrow BJ, Vadeboncoeur TF, Stolz U, Silver AE, Tobin JM, Crawford SA, Mason TK, Schirmer J, Smith GA, Spaite DW. (2013) The Influence of Scenario-Based Training and Real-Time Audiovisual Feedback on Out-of-Hospital

Cardiopulmonary Resuscitation Quality and Survival From Out-of-Hospital Cardiac Arrest. *Annals of Emergency Medicine* 62, 1: 47–56.

[11]Yang, CM, Wu, CH. (2005) The Situational Fatigue Scale: A Different Approach to Measuring Fatigue. *Quality of Life Research* 14 (5): 1357–62.

[12]Grant S, Aitchison T, Henderson E, Christie J, Zare S, McMurray J, and Dargie H. (1999) A Comparison of the Reproducibility and the Sensitivity to Change of Visual Analogue Scales, Borg Scales, and Likert Scales in Normal Subjects during Submaximal Exercise. *Chest* 116 (5): 1208–17.

[13]McDonald CH, Heggie J, Jones CM, Thorne CJ, Hulme J. (2013) Rescuer Fatigue under the 2010 ERC Guidelines, and Its Effect on Cardiopulmonary Resuscitation (CPR) Performance. *Emergency Medicine Journal* 30 (8): 623.
